# Bodily Practices and Healthy Bodies: Representations of Gymnastics in a Brazilian Women’s Magazine (1940–1950)

**DOI:** 10.3390/ijerph192315800

**Published:** 2022-11-27

**Authors:** Carolina Fernandes da Silva, Bruna Letícia de Borba, Cassiano Suhre da Rosa, Luiz Felipe Guarise Katcipis, Juliana Pizani, Janice Zarpellon Mazo

**Affiliations:** 1 Department of Physical Education, Sport Center, Federal University of Santa Catarina, Campus Trindade, Florianópolis 88040-900, Brazil; 2 State Educational System of Santa Catarina, Florianópolis 88035-901, Brazil; 3 Department of Physical Education, School of Physical Education, Physiotherapy and Dance Federal University of Rio Grande do Sul, Campus Jardim Botânico, Porto Alegre 90010-150, Brazil

**Keywords:** women’s health, body standards, gymnastics, women’s magazine

## Abstract

This research aims to understand the influence of bodily practices, especially gymnastics, in the construction of representations of a healthy body conveyed in a Brazilian women’s magazine in the 1940s and 1950s. We use records from the Jornal das Moças magazine for the analysis based on the theoretical and methodological assumptions of cultural history. The results show that gymnastics for women was linked to body maintenance and used as a tool for establishing a body standard, thus disciplining and shapingthe construction of women’s health concepts, determined by the aesthetic bias of that period: a slim body as an ideal standard of beauty and health.

## 1. Introduction

This study aims to understand the influence of bodily practices, especially gymnastics, on the construction of representations of a healthy body conveyed in a Brazilian women’s magazine in the 1940s and 1950s. The records from this magazine are analyzed in view of the theories of the body and methodological assumptions of cultural history. According to Chartier [1] (pp. 16–17), cultural history “has as its main objective to identify the way in which, in different places and times, a certain social reality is constructed, thought, given to be read”.

Bodily practices have approached the meanings of human movement actions beyond those that are thought of in their biological sense of adaptation. Aiming to advance in another direction and considering the current discussions about the complexity to conceptualize them [2,3,4], the direction of the present research follows an orientation permeated by the organization of the idea of techniques of the body by Mauss [5], which is characterized by the ways in which human beings, according to a tradition, perceive the use of their bodies. From this perspective, the body is understood as a social element, formed through the connections between individual and collective experiences of a singular time and space, in which the dialogues for building culture, and consequently the human world, are configured [6,7]. It is from the body that the possibilities of establishing interpretations about the world and others emerge, in a dialectical configuration defined in everyday life, which manifests itself through representations. These, in turn, are intrinsically related to a symbolic perspective, in which mediation is the central element of the perception of the phenomena experienced in the social scenario. Thus, the construction of values that configure public behaviors takes place, including a perspective woven by the creation of subjectivity [8].

Currently, the greater sharing of the space for the production of the discourse that guides the role of each individual in society, previously monopolized by the old-school, military and religious pedagogical forms, provides a course for changes, expanding the customs and institutions that lead to social rules of behavior. According to Vigarello [9] and Baumann [10], society has become more individualistic, where each subject is seen as an independent being and master of their destiny, having to identify themselves absolutely with their physical presence. This is transferred to the appearance of the body that has been projected more and more as an element of identity configuration, occupying a central place in the lives of individuals.

Appearance, behavior and silhouette are increasingly incorporated into personalities and personal particularities, so there is a more intimate relationship of existence from what I am to what my body is. Within this process, the changes of being a woman in contemporary times are involved with the changes that cross her body, as Vigarello [9] observed, when he found that dieting and losing weight constituted throughout modernity a normal condition for experiencing reality. It is important to highlight that the current lifestyle has given rise to a phenomenon related to health through the obesity epidemic, and that many of these people go through a process of loss of quality of life with the increase in mortality rate, with the need to seek ways to reverse this reality. However, it is necessary to reflect that some of these reflexes are not exempt from a perspective of political organization, production and consumption by which the government and capitalist systems prevailing in the current world revolve. The state’s health costs for an obese person almost double, bringing the perception that weight loss would be more a prevention of disorders and dysfunctions for their management system than for an aesthetic vision. The economic market, on the other hand, appropriates this fragility to turn body practices into a commodity developed in order to feed an aesthetic production of healthy body patterns [9,10].

The conceptions of women’s bodies and their representations within bodily practices can be presented as a set of elements that expose within them the influences of cultural formation, in which social concepts are enabled to build standards of fundamental structures of organization of life. To think about health in a context of transformation enables us to understand how it can reflect the intentions of a social situation and its ways of production. On the other hand, it also brings evidence of variations when considering power relations that stratify and hierarchize certain groups, discriminating them within the same space based on gender, race, economic conditions, religion and other aspects.

Throughout the structure of the social system of the 20th century, in the West, health was observed preponderantly through the bias of positivist rationality, thus denouncing incorrect techniques for the use of the body, composed essentially of biological matter in which the muscles were its driving machine [9]. At the same time, in Brazil, there was a change in the approach to sanitary problems, with an increase in the preventive nature, which was based on the establishment of controlling and regenerative measures inherent to the hygienist conception, which sought to establish a dominion over bodies in an attempt to foster care for the population by establishing new habits. Then, hygienism emerged as a way of structuring and instituting control over bodies, through the use of legislation, under the pretext of promoting the maintenance of the population’s health [11]. The health of children and women are now considered key factors in national regeneration. Thus, it is in the first decades of the 20th century that the health of this social segment has become part of public health policies [12].

At the same time, in Brazil, political projects on women’s education via body control were formulated, which had the support of the media press. During the period called Estado Novo (1937–1945), the Brazilian national project ruled by President Getúlio Vargas had women as an essential and central element for the betterment of the Brazilian people.

The increase in the working mass in precarious conditions of hygiene, health and housing, resulting from industrialization and urbanization, made women’s health a source of concern in several countries due to the accelerated growth of the world population. However, the emphasis remained on reproductive aspects. The woman that the state considered deserving attention was the one who was in the fertile period [12].

It is possible to find in the Brazilian press (newspapers and magazines) signs of the construction of an ideology of that time, including aspects related to modern civility and a new standard of aesthetics [13]. In the case of women’s magazines, despite not having among their communicative purposes the definition of rules and injunctions, they served as an instrument for the dissemination of social norms, since, by reading them, their public appropriated social rules. It is possible to notice, in their articles, changes and sustentation of practices and representations of the sociocultural conjuncture, such as a standardization of behaviors and role attributions of men and women [14,15].

Among the bodily practices assigned to women, gymnastics stands out compared to others. We can observe a representation of gymnastics different from those known today. Some of these representations are linked to the concept of body practices of the period, modernly called “gymnastics”, which, when directed to women, had specific objectives and constructions. That is, in that period, it was common to call “gymnastics” the body practices and/or physical exercises that were done daily, such as outdoor walks and morning stretching practices, among others [16]. Another important aspect is the search for modernity, which also encouraged the body practices that were in vogue in first world countries, developed by a model of physical culture, inspired mainly by Europe and Western America, such as the gymnastic methods. This model was based on an ideal of improving the physique and meant for the formation of a future with a strong and patriotic race [17].

During the 19th and 20th centuries, gymnastics developed in the West as an integral element of the modernization project, thus indicating a reorganization of the interpretation of reality. In this social dynamic, the conceptions of the human body were reconfigured by a distinct understanding of the meaning of relationships in the world [9,18]. In view of the above, we understand this as a new activity of cultural formation, as a set of symbols constructed and resignified by human beings to explain the world in which they live, translating an organization of reality [19]. In view of this, it is important to emphasize that for the theory of history, it is not appropriate to study an event from the perspective of right or wrong, invention or reality, or if in fact what was told existed that way. For historiography, it is interesting that if a vestige was consolidated in the present by its past, it is because in some way it contributed to the production of a meaning and thus had a connection with a network of social relationships that designated the conduct of life [20]. In the face of such a conception, it is necessary to decipher the verisimilitude of a past that is presented through its representations—their construction of a reality through the relationships among a multiplicity of discourses.

## 2. Materials and Methods

There is a diversification in the way content is analyzed based on the discourses in the documents that were gathered for this research, as well as the variety of format in which the information contained in them was recorded. Establishing perspectives that direct to a methodology is fundamental in order to focus on the reasons and the results consistent with the theoretical framework [21]). For Certeau [20], every historical operation, which involves the epistemological process of articulating a theory with the practice of the discipline, is referenced in the combination of a social place, scientific activities and writing. This study sought a place of socioeconomic, political and cultural production from a Brazilian women’s magazine in the second half of the 1940s and 1950s, with the purpose of understanding how its articles on bodily practices could be subjected, linked and rooted to the particularities of a representation of a healthy female body that was configured throughout the project of modernity.

Therefore, the analyses of the sources accessed for this historical research were submitted to the process of identifying whether there was correspondence between the information; that is, the narrated facts communicate with extratextual aspects of the period in order to situate the events in the midst of the interrelationships with the social phenomena that are formed in the modern conjuncture [19].

One of the main sources used by historians to identify traces of the past are periodicals, specifically newspapers and magazines, according to Barros [22]. For the present study, the magazine Jornal das Moças was selected as a source to identify the presence of bodily practices—specifically gymnastics—that evidenced the representations of a healthy body model present in the 20th century and modernity.

The reports of a single newspaper are not enough to explain the complex network that underlies the emergence of bodily practices linked to the health of the female body, but as the theory of cultural history demonstrates, they can constitute a historical source presenting evidence of a particular interpretation of the phenomenon in question [23]. The focus in this study was directed to find the common traits between the analyzed source and the social relationships developed in the formation of ideas to compose natural elements of a healthy body for women.

Jornal das Moças, a magazine published every two weeks in Rio de Janeiro that circulated in Brazil from 1914 to 1965, according to its founders, was designed to: “Cultivate, by illustrating, and at the same time delight in the charming spirit of the Brazilian women, to whom this magazine is dedicated, will be its, if not sole scope, at least its most lively and ardent concern” [24]. Five years after the first issue, in 1919, Jornal das Moças was already one of the most widely circulated magazines in Brazil [22]. However, the apex of sales reach occurred between 1945 and 1950, according to the Brazilian Institute of Public Opinion and Statistics (Instituto Brasileiro de OpiniãoPública e Estatística, IBOPE), which “reveals the popularity of this magazine: 1st place in the women’s press in 1945 (São Paulo) and 1st place among women’s weekly magazines during the 1950s ([in the states of] São Paulo and Rio de Janeiro)” [25].

The archive with all the issues of Jornal das Moças is available in the Digital Newspaper Library of the National Library of Brazil. However, only the issues published between 1945 and 1950 were consulted, since this wasthe magazine’s best-selling period, that is, with the widest audience reach, thus establishing the time period of the research. Using the search system available in the Digital Newspaper Library of the National Library of Brazil, with the term *ginástica* (gymnastics), we obtained 52 occurrences of this bodily practice within the chosen period. After this procedure of collecting the articles, in order to extract the information from the referred issues of the magazine, they were organized and stored as files in folders divided by themes and periods in common. The files were named by author, title of the report, date, issue of publication and page number, among other complementary information important for the elaboration of the source reference.

Of this total (52), we found that 29 occurrences were linked to health, both implicitly and explicitly. When it was implicit, the word “gymnastics” was not registered literally in the text, but it was in between the lines of the narrative that it referred to the health theme. In the explicit form, the terms “gymnastics” and “health” were registered directly in the same sentence in the text. After this step, we considered that six reports met the criteria established for the selection of information: the report had to contain aspects related to the use of some body technique in order to give meaning to human movement.

With this, the heuristic construction began, using two procedures approached by comparative history. Veyne [23] would use analogies to find how events that occurred worldwide or in other locations in the same period studied could reveal the same tendency to present the facts, and also the heuristic association with studies of historical conjunctures that preceded the period of the source studied, in which they may reveal a cultural environment of transformation of the source in connection with changes in social relations.

For Flick [21] (p. 295), coding terms “is often the combination of a good-quality analysis of some parts of the text and a preliminary, summary classification of other parts”. Text research involves coding the statements and narratives in developed categories, from the reading of these materials following specific procedures such as selecting the relevant parts, analyzing the data collection by identifying the origin of the documents, formally characterizing the material by reflecting how it was constituted, targeting what is expected to be interpreted from it and organizing it on a theoretical basis. In the thematic analysis method, the specific detail attributed to the research topic is deepened, and for this reason, it is relevant to demarcate to which degree of characterization these data areto be elevated. For the thematic analysis of the collected material, information was also grouped; in the case of this research, the sources were from a magazine and we focusedon identifying successive appearances that portrayed a standardized model and highlighting its meanings [21]. Thus, we used this method of analysis for six articles from the Jornal das Moças, seeking to interpret the texts in order to capture the meanings of the situational narratives in the researched sources.

## 3. Results and Discussion

### 3.1. Body, Beauty and Biopower: The Aesthetic Discourse of Jornal das Moças

According to Bauman [10], modernity comes from a continuous process of different modifications, and among them is the transition from traditionalism, which ceases to be the central foundation of its configuration, to a trend that will use symbolic structures within a concept of rationality. Through this process, forms of exercising power are organized to constitute techniques of control over bodies with the purpose of disciplining them for a certain social function that is expected of them throughout their lives.

Observing political life under the light of the articulation of modern states, this power was characterized by the consolidation of the promotion of social welfare through investment in life, and no longer through death as presented by the traditional sovereignty regime, forming what Foucault [6] (p. 131) called *biopower* with “the administration of bodies through the calculating management of life”. These movements would promote new breakdowns of the different spheres of human social life, thus causing the accentuation of more demanding and precise rules of body appearance, making body fat, for example, also a reflection of personality and even associated with ways of ordering one’s thoughts [9].

Among the devices that bring society closer to a new world of modernities and new social ruptures, in this research we focus on women’s magazines. Such magazines began to be published at the end of the 19th century with the intention of catering to the minds of readers’ new perceptions of the world and, consequently, new representations of health of women’s bodies. It is worth recalling the statement by Barbosa et al. [26] (p. 24): “The history of the human body is the history of civilization. Each society, each culture acts on the body by determining it, […] creating its own standards.”

In the mid-1940s, the entire world was undergoing a radical transformation, including the reorganization of the center of economic power among nations with the end of the Second World War. The United States of America and the Soviet Union would form a bipolar order of command over government systems, through which structures would be set up to shape advantages that could favor their models of life management. It was during this period that the United Nations (UN), United Nations Children’s Fund (UNICEF), Food and Agriculture Organization of the United Nations (FAO/UN), United Nations Educational, Scientific and Cultural Organization (UNESCO), International Labor Organization (ILO) and World Health Organization (WHO) were created, which would serve as a legal, political and ideological instrument for an internationalism necessary for the interest of free-investment capitalism, enforcing a certain type of planning for postwar life [27].

Distinct cultures are propagated among countries through processes of transference and reception of body standards. An example of this cultural dynamic was evidenced in the article from the Jornal das Moças, whose title was *3 Meses de Ginástica em Quatro Páginas* (Three Months of Gymnastics in Four Pages) [28], which showed support and incentive for the diffusion of a model of physical culture based on an ideal of physical improvement, with gymnastics as a practice for the creation of a strong and patriotic race. These precepts had roots in countries such as France, Germany and the United States [17]. In anexcerpt from that article, it says: “We must confess that the benefits of gymnastics are well known. You admit, for example, the need for a famous quarter of an hour in the morning… But from there to really take advantage of it goes a long way! This is what we are talking about here; is no new method: it is a new understanding of physical culture” [28].

Moreover, in the excerpt mentioned above, we can identify a relationship between the meanings proposed for physical culture within the manifestation of the concept of culture built within the European context during the 18th and 19th centuries. In that period, the term was configured as a representation linked to human development, which soughtalignment with the aspect of civilization from a historical reconstruction, which denoted shades of the advance of humanity to greater freedom from the bonds of irrationality [29]. In the case of Brazil, physical culture in the 1940s and 1950s was seen as part of a process of describing a certain intellectual, spiritual and aesthetic development, whose purpose wasto qualify the body within a conception of evolution. With this, physical culture received the characteristics of human production that enabled the classification of groups based on the organization of degrees of civility to support the differences between them, following the idea of a greater psychic control over movement [29,30].

In the Jornal das Moças magazine, the expression “physical culture” appears in the texts since the 1930s and maintains its incidence until the beginning of 1960, referring to the idea of the mind–body dichotomy, indicating the importance of understanding that in order to achieve proper mental development, one must exercise the body. Thus, exercise through gymnastics would be a way to improve the power of psychic control over movement, which, when performed correctly, would generate alignment with the expected aesthetic standard; the correct posture of the body is perceived by the symmetry of its parts. Furthermore, in the case of women, exercise through gymnastics should express a greater control over the body (understanding that the psyche is part of that body) and not the demonstration of some ability.

Physical culture is related to a specific knowledge of how to control the body by directing its movements to the designs of modern society, and gymnastics is one of the composing elements. These notions cross over into the conceptions of bodily practices aimed at women, presenting themselves as a tool for maintaining beauty, which can be conquered by willpower, rigor and discipline [31]. Among the practices presented in the articles, we can find gymnastics for the hands, the face, the hips, and even the lips, as in the excerpt: “Let us not be alarmed, readers! The lips also need to be exercised like the limbs, like the muscles, like the hands, so that the straight gymnastics of the mouth keep them fresh. Correct mouth gymnastics keeps the lips in perfect line.” [32]. These propositions are often found in the print, in which bodies are presented as plastic, moldable matter, amenable to being scrutinized and treated separately [31].

However, as part of the culture, the construction of images of what it is to “be feminine” appears in different spaces and times under different forms, strategies and discourses. According to Pinsky [25,33], models of femininity were consolidated from the early twentieth century until the early 1960s, and we can observe a proximity between performing bodily practices and the construction of the elements of these representations. According to the Jornal das Moças magazine, it is through conceptions of beauty that these representations are also constructed, where the gestures are reproductions of femininity, as in the report that recommends gymnastics for the hands: “A female woman is one who, without escaping her personality, is obliged to cultivate elegance and beauty […] and to take care of herself, never presenting herself with an aspect of slovenliness, which makes the man lose his attraction for her. A moderately vain woman is more attractive and more of a woman than others […] In cities, it is customary to judge women by the hand. They must therefore give him special treatment. It’s a simple matter of willpower […] hand gymnastics is essential” [34].

The concern with a healthy appearance was surrounded by having a youthful appearance, which could be maintained, acquired and corrected through gymnastics, also with the promise of a rejuvenating body, as can be seen in this publication on gymnastics for the face: “Five minutes a day of this highly beneficial gymnastics, in every way, completes this wonderful new treatment, which makes the face fresher, younger, giving it hardness. and normalizing the secretions of the skin, which gives the features and muscles the necessary elasticity to perform more graceful gestures” [35]. In this way, the magazine explored all body segments in the search for the formatting of women’s models that served an ideal of modernity. As the historian Sevcenko [36] points out, changes in the behavior patterns of individuals imposed by the preeminence of machines, by the engineering of flows and by the culture of consumption provoked a change in the values framework of society. People began to be evaluated no longer by their internal and personal qualities, but by the way they dressed, by the symbolic objects they displayed, by the way they behaved, in the modeling of their body, in their education and in the improvement of their expression by external codes. In other words, your social visibility and your power of seduction are directly proportional to your purchasing power. Barros [37] states that such recommendations targeted the new Brazilian women, who should be active outdoors and exercise their bodies.

### 3.2. Fatness and Thinness in Review: The Body as a Social Distinction

History identifies that the traditional requirement of beauty refers to the description of a slender, perfect body, and new artifices can be used to correct its flaws [37]. In light of this, a series of observations regarding procedures performed by women in order to control aesthetic standards were published, with the purpose of maintaining elegance: “Women are most impressed by their silhouette. For her, losing or gaining a few grams is a real nightmare! That’s why she keeps looking for a scale, regretting if her weight increases or decreases” [37]. In the same passage of the article, the author adds greater emphasis to a culture of concern with weight with the following statement: “It is not so difficult to maintain a normal, regular body. Just follow a proper diet, practice proper gymnastics […]” [38]. Thus, the magazine indicated that a thin body would be the desirable “normal and regular body”, which represented health and beauty in that period.

Sometimes, the representations of health and beauty appear as synonyms in the reports, which leads women to a total dissatisfaction with their bodies. Yoga, for example, is represented by the magazine as a practice that leads to “conservation of health and beauty” [39], (p. 46). According to Vigarello [40], aesthetics becomes ethics. The ugly ones were in disgrace, until the 20th century speeches of stimulus to consumption, with the maintenance of health as a justification, affirmed: all women can be beautiful. Moreover, bodily practices can be a propelling tool to achieve health and ward off unwanted ugliness. The prevention of ugliness, in the context of Brazilian society, transforms and translates the lack of beauty in terms of illness [41].

Throughout the 19th century, Western medicine sought its organization in the face of modernity through the precepts of the paradigms of a positivist science, in which knowledge about the body emerged from clinicopathological anatomy. Thus, the human body came to be constituted as if by right of biological matter, thus being a space of origin and distribution of disease [42]. From this idea, the perception of the existence of a body normality was built, and the physician assumed an important role in observing a healthy human organism standard, having the power to prescribe treatments that involved weight loss processes. Weight loss is part of a set of disciplinary techniques that compose hygiene practices of the individual with the intent not only to protect the body from illness, but to fill it with a moral essence [9,42].

Therefore, it is noticeable that there is a growing concern for women’s thinness as a health component, but also as an aesthetic element revealed by the fashion magazines that address getting fat as one of the greatest fears of women. The female body has been required to occupy public spaces by being endowed with mobility through a slim silhouette, which took into account thin, muscular, fat-free limbs with tapered lines [9]. This is because modernity is wrapped up in the idea of lightness, flexibility and agility, thus increasing body exposure with the reduction of clothing and with the new bodily practices that emerged, such as bathing in the sea, extending an aesthetic concern to other body parts that were previously covered. As a trend from this orientation, waist circumference measurements were introduced in medical appointments, the relations of height and weight values were explored, and thus, ideal standards for each gender were created [9].

Bodily practices have become the main tool for achieving an ideal healthy body for women in Brazil, with the encouragement of the press. In light of this, in the 1940s and 1950s paradigms shifted about women’s bodies. Performing bodily practices was a requirement for those considered modern high-class women entering life [13], a behavior that started to influence other women. Jornal das Moças had as its target audience the “Brazilian women”; however, it becomes evident that the aspects that represent the profile of this audience came from the hegemonic social values of that time, which means white and thin women, which stood out as ideals of a maternal and wife scope. It is noticeable that there are several modifications in the standardized body project, which is closely related to the gender ideal that is configured and categorized according to the interests of social relations. Women are included and have their representation defined according to their aspect of historical location, through economic, political and ideological relations in social structures, generally under the power of men [43].

In an article entitled “Código da Linha” (Code of the Line) [44], Jornal das Moças puts forth an analogy using traffic signs to inform different weight codes and concerns. Within the content of the article, there are mentions of this control through numbers, for example, in a sign entitled “Double Defense” there is the following description: “A few superfluous pounds are enough for you to “double” your weight… and appear to double your age (metaphors cruelly true). A small lacquered scale should find a place in your bathing room. It is the only way to follow, day by day, the increase of your weight, and to make it stop in time” [44]. On another sign, still on the same page, with the title “Stopping Prohibited”, the magazine informs about the importance of keeping the body in movement and uses numbers to provide rhythm and control to the gymnastics, as can be seen in the following text fragment: “When jumping out of bed, immediately… one, two, three the gymnastics” [44].

In this scenario, the applied concept of “female gymnastics” comes from the medicalhygienist perspective. In this sense, gymnastics has as its guiding principle, in its conception, the strengthening of women’s bodies, because it was considered that strengthened women would have male children endowed with strength, who would be responsible for building and protecting the country [45]. However, this was without losing the feminine traits, which were obtained through the suggestions published in women’s magazines of that period, once again highlighting the media influence in the orientation of what would or would not be necessary to achieve through bodily practices. According to Mendes [46], gymnastics has contributed to the intertwining of the concept of health with the concept of aesthetics within a reductionist perspective, reinforced by certainmedia scenarios, and contributing to the construction of a healthy body standard for Brazilian women.

However, this discourse on body standards, present in Jornal das Moças, socially builds the idea that being overweight means assuming the failure of evolution, thus pushing women away from the idea of being in progress in life, prevented from experiencing the world due to having a silhouette that goes against the dominant culture and thus making them strangers even to themselves [9]. In this sense, gymnastics ends up being a tool that induces the shaping of an aesthetically beautiful, attractive and fat-free model of women’s bodies.

Since its beginning, the 20th century has been marked by many discussions about a possible universal concept of health. Influenced by the organizational policies of nations that sought in the greater intervention of the state a way to systematize control over the bodies of the population, in the idea of construction and preparation of knowledge aimed at the insertion of individuals in the new dynamism of modern life, the political scenario of the 1940s experienced the effervescence of these ideas. This is because at the end of this decade, the World Health Organization (1948) was established, which would define health as “the state of complete physical, mental and social well-being and not merely the absence of disease”, thus raising its scope in the social structure to a level not yet understood by many nations. This concept certainly promoted a reorganization of the discipline over the bodies, by placing health in an even more vigilant, or even unattainable level, in which bodily practices are the living manifestation of the transformations.

## 4. Conclusions

The representation itself of a healthy female body that the bodily practices were intended to create would not be historical if it were not noticed as an action of deviation of meaning, which only becomes possible when articulated to a social place and scientific operation related to cultural models or contemporary theorists. Therefore, the writing that articulates the facts of the past is given by the presence in the present of problems that lead to question the reasons for which the reality is being juxtaposed.

The evidence found in Jornal das Moças suggests that gymnastics in the analyzed period had different representations, but when aimed at women, it was linked to the maintenance of bodies and beauty. In addition, it was used as a tool to achieve a body standard, aiming to regulate, standardize and discipline women’s behaviors. Therefore, we consider that gymnastics played an important role in the construction of women’s health concepts in Brazil in the 1940s and 1950s.

Every narrative comes from a context, seeks bases in ideas and elements that pursue specific views and perspectives of a time, builds symbolic connections that are convenient as a way of interpreting reality and seeks meaning from the previous belief that the past was exactly what is presented in the present, because no human discourse arises out of nowhere and is always surrounded by intentions that circulate subjectivities. For Foucault [47], the dispute over powers that influence human decisions are marked by a microphysics in which participation takes place under the influence of different structures dissolved throughout the social fabric. Therefore, although there are general trends articulated by the state to relate body practice to the representation of a healthy body, it is possible to capture at the other end the specificities that each locality assumes, when appropriating the disseminated knowledge.

In light of the above, it is possible to understand how bodily practices, in particular gymnastics, created representations of healthy bodies in the magazine Jornal das Moças through the aesthetic bias of that period: a slim and beautiful body. Conceptions of healthy bodies were built through the narratives of Jornal das Moças in the country. After all, when the women’s press was established in Brazilian society, it became an important vehicle of communication and injunctions that disciplined and educated women both culturally and socially.

A limitation of this study could be the ability to develop the search for the particularities of the phenomenon studied within the Brazilian context, because for this it would be important to select other sources that could present the modifications and preferences that were formed through adialogue with the local culture. This is a gap that this research leaves, but that does not disqualify the contribution made by this study to research on women, gymnastics, health and body practices, since it is in line with the rigor of historical research that, according to Veyne [23], takes place at the level of criticism with the purpose of becoming more aware of the reality in which we live.

Among the gender issues that we can address in future studies is the debate that, when analyzing the representations of the Jornal das Moças magazine, we are talking about a reality of hegemonically white, literate upper-class Brazilian women. This is because the target audience of the magazine was these women. The phenomena that permeate the issues of black and lower middle-class women, for example, are different, and may be presented differently in reports, constituting an interesting aspect for analysis and a deepened investigation.

The inductions in the disseminated texts proposed an understanding about the achievement of the ideal body, and ways in which women could reach this “model,” placing discipline, the will to achieve this goal and the responsibility of maintaining beauty and health as key elements. We can conclude that the notions of body formed from the characteristics linked to gymnastics in the cultural-historical perspective of the period weremixed and wentthrough a diversity of scenarios, in which both the rhetoricand narratives acted directly on women’s bodies and helped to construct gender stereotypes.
